# Giant Coronary Aneurysm Causing Ostial Occlusion of Coronary Artery by Mass Effect: A Case Report

**DOI:** 10.7759/cureus.16280

**Published:** 2021-07-09

**Authors:** Vanlalmalsawmdawngliana Fanai, Amit Malviya, Animesh Mishra, Donboklang Lynser, Tony Ete

**Affiliations:** 1 Cardiology, North Eastern Indira Gandhi Regional Institute of Health and Medical Sciences, Shillong, IND; 2 Radiology, North Eastern Indira Gandhi Regional Institute of Health and Medical Sciences, Shillong, IND

**Keywords:** giant coronary artery aneurysm, acute myocardial infarction, thrombosis, coronary anomaly, computed tomography, cardiac imaging, mechanism, diagnostic methods, invasive coronary angiography, management

## Abstract

Coronary atherosclerosis can rarely lead to complications like giant coronary aneurysm (GCA), and acute myocardial infarction (AMI) due to thrombosis in the GCA is even rarer. Multimodality imaging is preferred over relying solely on selective coronary angiogram in such cases due to the limitations of invasive coronary angiogram in visualizing thrombosed aneurysms. We report a rare case of a patient with ST-elevation myocardial infarction caused by ostial occlusion of a right coronary artery (RCA) due to mass effect created by thrombosis in a GCA, thereby highlighting a mechanism of AMI that has not been previously described in GCA. Multimodality imaging led to the correct diagnosis and detection of the underlying mechanism, which had been completely missed by invasive coronary angiography (ICA). We also discuss the utility of multimodality imaging in such cases.

## Introduction

Aneurysmal dilatation of coronary artery and giant coronary artery aneurysm (GCA) are rare findings during angiography [1,2]. There is no clear definition of GCA, but in the context of Kawasaki disease, large or giant aneurysms are defined as those with an internal lumen diameter >8 mm [3], and some experts consider atherosclerotic aneurysms >2 cm as GCA [4]. Although invasive coronary angiography (ICA) is the gold standard imaging technique in such cases, it may fail to detect coronary artery aneurysm (CAA) in the presence of luminal thrombi. In this report, we present a case of GCA involving left anterior descending artery (LAD) and right coronary artery (RCA) presenting as acute myocardial infarction (AMI) due to the occlusion of RCA ostium caused by the mass effect of thrombosed GCA of RCA. In this case, multimodality imaging led to the correct diagnosis and the detection of the underlying mechanism, which had been completely missed by ICA. To the best of our knowledge, GCA causing AMI due to mass effect has not been previously reported in the literature.

## Case presentation

**Figure 1:**
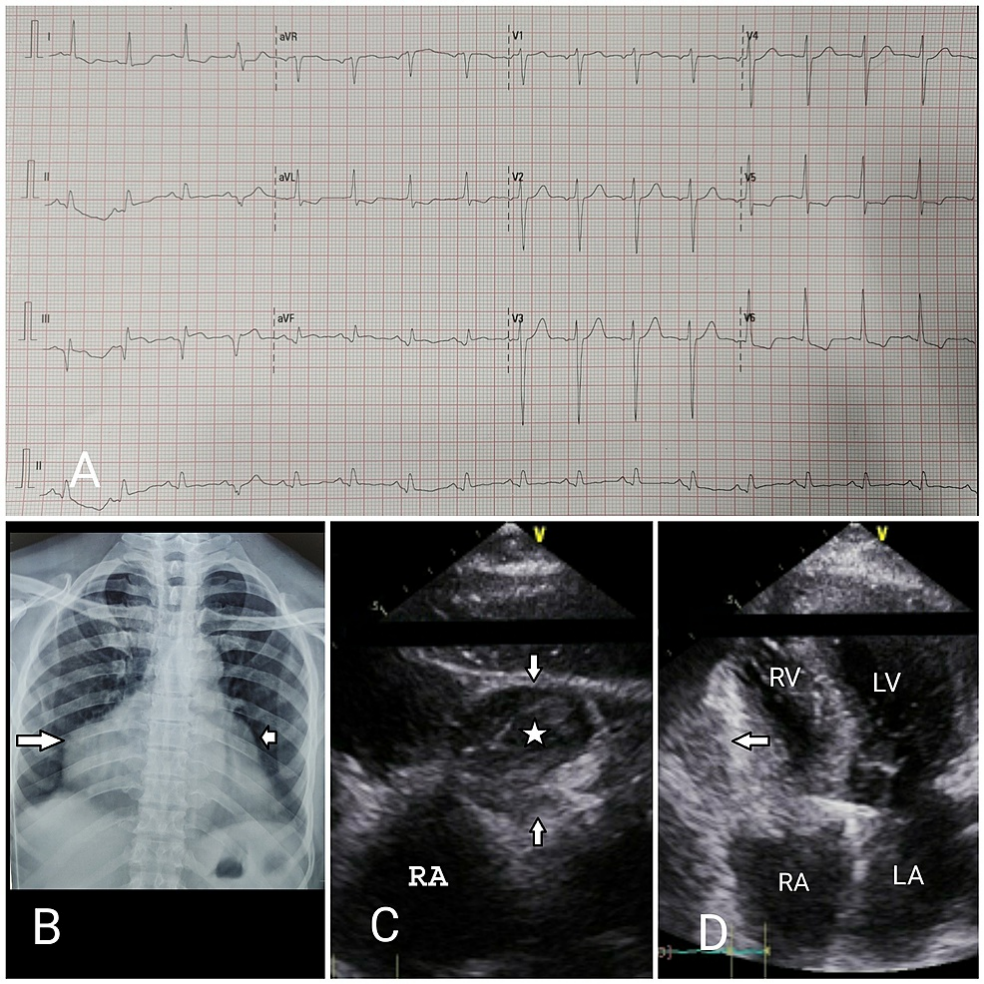
Noninvasive diagnostic images A: Electrocardiogram showing sinus rhythm with ST elevation in lead III and aVF along with reciprocal ST depression in lead I, aVL, and V5-V6. B: Posteroanterior view of chest X-ray showing cardiomegaly with a focal bulge from the right (long white arrow) and left cardiac contours (short white arrow) consistent with aneurysms from the right and left coronary arteries respectively. C: Echocardiographic image: modified subcoastal view demonstrating a well-circumscribed cystic mass measuring 74 x 60 mm (white arrow) with heterogeneous echogenic content (white star). D: Echocardiographic image: apical four-chamber view showing extra-cardiac mass (white arrow) compressing RV RA: right atrium; RV: right ventricle; LA: left atrium; LV: left ventricle

A 60-year-old male non-smoker, non-diabetic, and normotensive patient was referred to our center with a history of retrosternal chest pain and dyspnea [New York Heart Association (NYHA) class IV] for two days. The patient had a history of exertional dyspnea and angina (NYHA class II) for the past year, but he was neither on any sort of medication and nor had consulted any physician for the same. The patient denied any past history of fever, joint pain, limb claudication, trauma to the chest. He was a farmer by occupation, and prior to this visit, there was no history of any medication or procedure done on him. On physical examination, his blood pressure was 100/70 mmHg, pulse was 100/minute, and regular in rhythm. The rest of the cardiovascular examination was unremarkable. An electrocardiogram at admission showed sinus rhythm with ST elevation in inferior leads (lead III/aVF) along with reciprocal ST depression in lead I, aVL, and V5-V6 (Figure 1A). The cardiac biomarkers were significantly elevated. Initial imaging with chest X-ray revealed cardiomegaly with enlarged right heart border (Figure 1B), and 2D-echocardiogram demonstrated inferior wall hypokinesia with mildly reduced left ventricular systolic fraction and a well-circumscribed cystic mass adjacent to the right ventricle (Figures 1C, 1D). The routine biochemistry findings are provided in Table 1.

**Table 1:** Routine biochemistry at admission HBsAg: hepatitis B surface antigen; HCV: hepatitis C virus; IgM: immunoglobulin M; HIV: human immunodeficiency virus; VDRL: venereal disease research laboratory

Investigation	Value
Hemoglobin (g/dl)	13.6
Total leukocyte count (per microliter)	6700
Platelets (per microliter)	270 x 10^3^
Random blood sugar (mg/dl)	98
Urea (mg/dl)	50
Creatinine (mg/dl)	1.1
Uric acid (mg/dl)	6.6
Sodium (mmol/L)	138
Potassium (mmol/L)	4.34
Triglyceride (mg/dl)	86
Total cholesterol (mg/dl)	86
High-density lipoprotein (mg/dl)	16.5
Low-density lipoprotein (mg/dl)	69.6
Total bilirubin (mg/dl)	0.7
Aspartate aminotransferase (Unit/L)	105
Alanine aminotransferase (Unit/L)	50
Creatine kinase-MB (Unit/L)	154
High-sensitivity troponin I (pg/ml)	1315 (normal range: 0-17.5)
HBsAg	Nonreactive
Anti-HCV IgM	Nonreactive
HIV 1 and 2	Nonreactive
VDRL test	Nonreactive
Blood culture	Sterile
C-reactive protein (mg/L)	7

**Figure 2:**
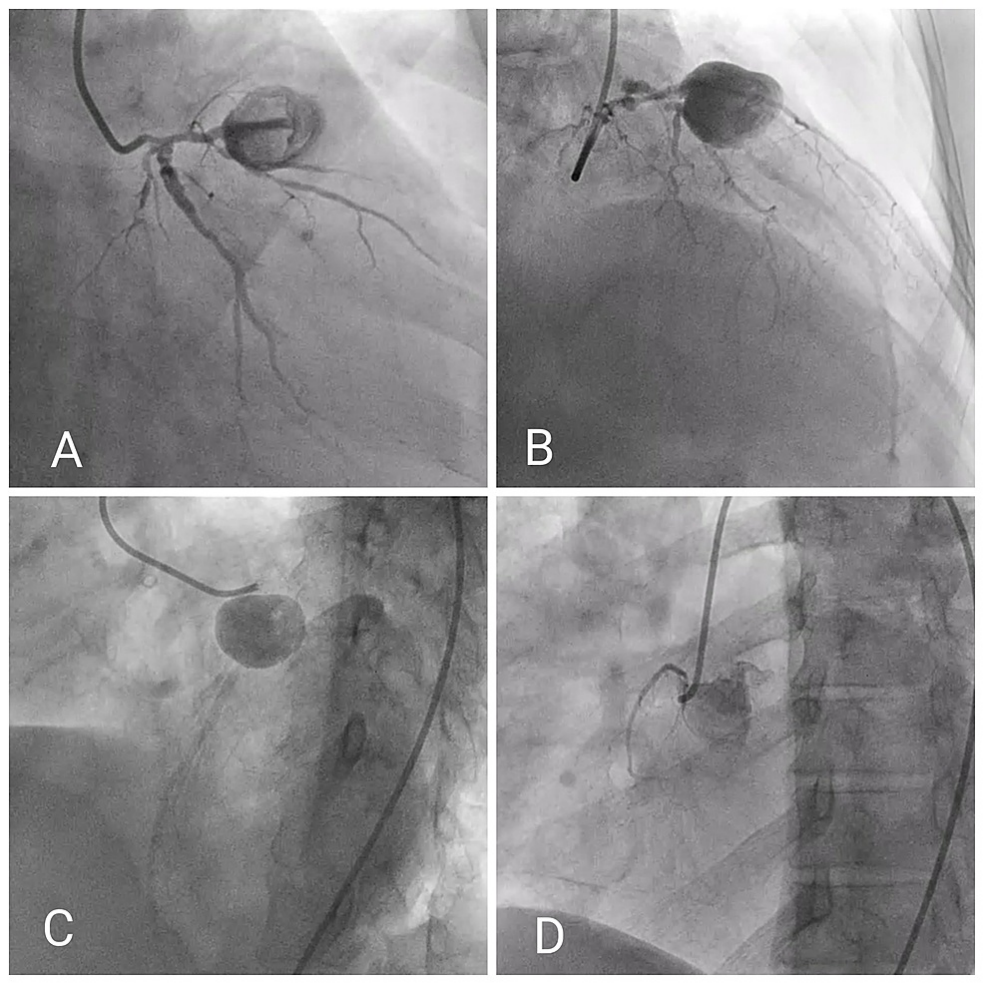
Angiogram of coronary arteries A: RAO caudal view showing significant stenosis of left main coronary artery and GCA at proximal LAD artery with freely flowing contrast material within the aneurysm. B: RAO cranial view showing GCA at proximal LAD artery with TIMI flow II distal to the aneurysm. C: LAO cranial view showing similar lesion as described above with TIMI flow II distal to the aneurysm in LAD. D: LAO view of nonselective contrast injection at the aortic root demonstrating total occlusion of ostial-proximal RCA and conus branch arising from a separate ostium GCA: giant coronary aneurysm; LAD: left anterior descending; LCX: left circumflex artery; LAO: left anterior oblique; RAO: right anterior oblique; TIMI: Thrombolysis in Myocardial Infarction

Angiogram of the left coronary system (Figures 2A-2D; Videos 1, 2) showed significant left main stenosis with a GCA arising from proximal LAD (Figures 2A-2C) with Thrombolysis in Myocardial Infarction (TIMI) grade II flow distal to the aneurysm. Diffuse significant lesions in the proximal left circumflex artery and its major branches were also observed.

**Video 1:** RAO cranial view of coronary angiogram demonstrating freely flowing contrast material within GCA at proximal LAD artery and TIMI grade II flow distal to the aneurysm GCA: giant coronary aneurysm; LAD: left anterior descending; RAO: right anterior oblique; TIMI: Thrombolysis in Myocardial Infarction

**Video 2:** LAO caudal view of coronary angiogram demonstrating significant stenosis involving left main coronary artery and proximal LCX artery LAO: left anterior oblique; LCX: left circumflex artery

The ostium of RCA could not be engaged selectively despite several attempts, and a nonselective angiogram revealed occlusion of RCA from the ostium with mild disease in the conal branch of RCA arising from a separate ostium (Figure 2D, Video 3).

**Video 3:** Nonselective coronary angiogram at aortic root demonstrating occlusion of RCA from the ostium with mild disease in the conal branch of RCA arising from a separate ostium RCA: right coronary artery

**Figure 3:**
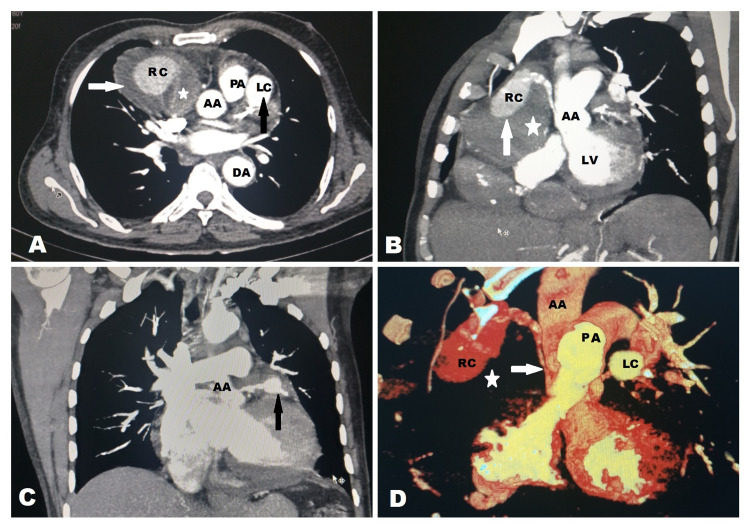
CECT of the thorax showing aneurysms arising from both right coronary artery and left anterior descending artery A: CECT in axial sections showing aneurysm (80 x 60 mm) (arrows) arising from the RCA (RC) with mural thrombus (star) and left LAD (30 x 30 mm) (LC) without mural thrombus (black arrow). Note the ascending aorta (AA), descending aorta (DA), and main pulmonary artery (PA). B: Maximum intensity projection (MIP) CECT in the oblique coronal plane showing the RCA arising from ascending aorta (AA) with aneurysm (RC) with mural thrombus (star). Note the ascending aorta (AA) and left ventricle (LV). C: MIP CECT in the coronal section showing the LAD arising from the ascending aorta (AA) with aneurysm (black arrow). D. Volumetric reconstructed CT scan showing the aneurysm from the RCA (RC) and LAD (LC). Note the mass effect on the proximal RCA (white arrow) at its origin from the ascending aorta (AA) due to aneurysm with mural thrombus component (white star) CECT: contrast-enhanced computed tomography: RCA: right coronary artery; LAD: left anterior descending

Subsequently, contrast-enhanced CT (CECT) of the thorax and coronary CT revealed a partially thrombosed giant aneurysm (80 x 60 mm) arising from the proximal RCA (Figure 3), compressing the proximal segment of RCA against the aortic wall and causing occlusion from the ostium and a giant saccular coronary aneurysm (30 x 30 mm) arising from the proximal segment of LAD. No other abnormality was noted in the aorta and other major vessels, nor any signs of vasculitis were detected.

The patient was counseled on the need for surgical intervention for his treatment, but he opted for medical management only. He was treated with guideline-directed medical therapy including anticoagulation and was discharged in a hemodynamically stable condition. The follow-up data was not available at the time of writing this case report.

## Discussion

Giant aneurysms are very uncommon and are found in only 0.02-2% of the general population [5]. The majority of GCA in adults is attributed to atherosclerosis. Other causes of GCA include congenital malformation, Takayasu's arteritis (<40 years of age), connective tissue disease, other forms of vasculitis, infections, drugs, chest trauma, cardiac lymphoma, and trauma during angioplasty [6]. The most common affected artery is RCA, and concomitant involvement of other coronary arteries is uncommon [7].

The clinical presentation of GCA may range from asymptomatic to life-threatening complications. Although rare, an aneurysm could undergo thrombotic changes and may lead to AMI, but the exact incidence of AMI in GCA remains unknown [8]. Forte et al., in a study from Italy, have reported that 66.7% of CAA developed intraluminal thrombosis, out of which only 22.3% presented with atypical chest pain [9].

The sensitivity of multi-slice CT coronary angiography (MSCT-CA) to detect CAA is nearly 100% [10]. Besides providing information about the morphology of aneurysm, MSCT-CA helps to capture complex anatomy and detect intraluminal thrombi. Furthermore, maximum intensity projections (MIP), curved multi-planar reformations (c-MPR), and 3D volume clearly display the anatomical relationships of the aneurysm to the surrounding structures [9]. Therefore, it is wise and logical to incorporate multimodality imaging, especially CT scan, when GCA is detected in ICA.

Currently, there are no proper guidelines regarding the management of AMI caused by GCA. Treatment options include percutaneous coronary intervention (PCI) using a covered stent and surgical intervention on the background of medical therapy [11]. One study recommends surgical treatment for all CAAs that are >3 cm because of the risk of rupture [10]. The Coronary Artery Aneurysm Registry (CAAR) [12], the largest multicentre registry (including >1500 patients) of such cases, found the mortality and major adverse cardiac events (MACE) rates to be 15.3% and 31%, respectively, in such cohorts, and hence timely treatment is of utmost importance.

The main aim of the current case report is to highlight the importance of multimodality imaging in the diagnosis and characterization of GCA. Although selective invasive coronary angiogram remains the gold standard to diagnose GCA, an initial noninvasive test may provide a clue to its diagnosis by the detection of mass-like appearance on chest X-ray and echocardiogram. Moreover, the major limitation of ICA is the failure to detect aneurysms in the presence of intraluminal thrombi and its relationship to the surrounding structures, as it is a luminogram only [8]. In our case, thrombosis of RCA aneurysm led to compression of the proximal segment of RCA due to mass effect causing AMI. This was missed by ICA, which gave an impression of ostial occlusion. Also, the RCA aneurysm was not visualized on ICA.

## Conclusions

GCAs are very uncommon and are found in only 0.02-2% of the general population. AMI caused by thrombus formation is an uncommon complication of GCA. We described a case of AMI due to compression of a coronary artery due to mass effect, which has not been described before. Noninvasive tests may provide a clue to the diagnosis of GCA, and relying solely on invasive selective coronary angiogram may fail to detect thrombosed GCA; hence, it is prudent to incorporate multimodality cardiac imaging when GCA is encountered. Besides providing information about the morphology of aneurysm, MSCT-CA also helps to capture complex anatomy and detect intraluminal thrombi.
